# SELF-REPORTED WALKING PACE AND ALL-CAUSE MORTALITY AMONG CANCER SURVIVORS IN THE NIH-AARP DIET AND HEALTH STUDY

**DOI:** 10.1093/geroni/igz038.1423

**Published:** 2019-11-08

**Authors:** Elizabeth Salerno, Pedro Saint Maurice, Erik Willis, Loretta DiPietro, Charles Matthews

**Affiliations:** 1 National Cancer Institute, Rockville, Maryland, United States; 2 George Washington University, Washington, District of Columbia, United States

## Abstract

We examined the association between self-reported walking pace and all-cause mortality among cancer survivors in the NIH-AARP Diet and Health Study. Participants included 30,110 adults (Mage=62.4+/-5.14 years) diagnosed with cancer between study enrollment and follow-up, when they self-reported walking pace. Individuals were followed until death or administrative censoring in 2011. We estimated the hazards ratios (HR) and 95% confidence intervals (CI) for walking pace and all-cause mortality adjusting for age, sex, race, BMI, health status, physical activity and cancer type. Cancer survivors reporting faster walking paces had significantly reduced mortality risk. Relative to those reporting an ‘easy’ walking pace, walking at a ‘normal,’ ‘brisk,’ or ‘very brisk’ pace was associated with significantly lower risk: [HR=0.74 (0.70,0.78)], [HR=0.66 (0.61,0.71)], and [HR=0.73 (0.60,0.89)], respectively. Being ‘unable to walk’ was associated with 30% increased mortality [HR=1.30 (1.15,1.46)]. These findings provide novel support for the association between self-reported walking pace and survival after cancer.

